# Metal‐Specific Biomaterial Accumulation in Human Peri‐Implant Bone and Bone Marrow

**DOI:** 10.1002/advs.202000412

**Published:** 2020-08-03

**Authors:** Janosch Schoon, Bernhard Hesse, Anastasia Rakow, Melanie J. Ort, Adrien Lagrange, Dorit Jacobi, Annika Winter, Katrin Huesker, Simon Reinke, Marine Cotte, Remi Tucoulou, Uwe Marx, Carsten Perka, Georg N. Duda, Sven Geissler

**Affiliations:** ^1^ Julius Wolff Institute Charité – Universitätsmedizin Berlin Berlin 13353 Germany; ^2^ Berlin Institute of Health Center for Regenerative Therapies Berlin Institute of Health Berlin 10178 Germany; ^3^ Berlin‐Brandenburg School for Regenerative Therapies Charité – Universitätsmedizin Berlin Berlin 13353 Germany; ^4^ Xploraytion GmbH Berlin 10625 Germany; ^5^ European Synchrotron Radiation Facility Grenoble 38000 France; ^6^ Center for Musculoskeletal Surgery Charité – Universitätsmedizin Berlin Berlin 10117 Germany; ^7^ Department of Materials Science and Engineering Institute of Materials Science and Technologies Technische Universität Berlin Berlin 10623 Germany; ^8^ TissUse GmbH Berlin 13347 Germany; ^9^ Endocrinology and Immunology Department Institute for Medical Diagnostics Berlin 12247 Germany; ^10^ CNRS Laboratoire d'archéologie moléculaire et structurale LAMS Sorbonne Université Paris 75005 France

**Keywords:** arthroplasty, bone marrow, metal exposure, nanoparticles, synchrotron radiation

## Abstract

Metallic implants are frequently used in medicine to support and replace degenerated tissues. Implant loosening due to particle exposure remains a major cause for revision arthroplasty. The exact role of metal debris in sterile peri‐implant inflammation is controversial, as it remains unclear whether and how metals chemically alter and potentially accumulate behind an insulating peri‐implant membrane, in the adjacent bone and bone marrow (BM). An intensively focused and bright synchrotron X‐ray beam allows for spatially resolving the multi‐elemental composition of peri‐implant tissues from patients undergoing revision surgery. In peri‐implant BM, particulate cobalt (Co) is exclusively co‐localized with chromium (Cr), non‐particulate Cr accumulates in the BM matrix. Particles consisting of Co and Cr contain less Co than bulk alloy, which indicates a pronounced dissolution capacity. Particulate titanium (Ti) is abundant in the BM and analyzed Ti nanoparticles predominantly consist of titanium dioxide in the anatase crystal phase. Co and Cr but not Ti integrate into peri‐implant bone trabeculae. The characteristic of Cr to accumulate in the intertrabecular matrix and trabecular bone is reproducible in a human 3D in vitro model. This study illustrates the importance of updating the view on long‐term consequences of biomaterial usage and reveals toxicokinetics within highly sensitive organs.

## Graphical abstract

Spatially micron‐ and nanoresolved synchrotron X‐ray fluorescence analyses of peri‐implant cancellous bone reveal specific distribution and accumulation patterns of metals released from alloys frequently used in knee and hip arthroplasty. These new insights of the local toxicokinetics of metallic debris prove that cobalt, chromium, and titanium exposure also occurs behind the insulating implant membrane, in the bone and bone marrow.

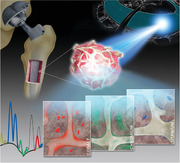

## 1 Introduction

Modern arthroplasty implants enable painless mobility and ensure significant improvement of life quality. A stable integration of the artificial joint in the peri‐implant mineralized tissue is crucial for its success. Materials used in arthroplasty are of multi‐elemental composition to ensure long‐term mechanical stability of the implant.^[^ ^1^ ^]^ Cobalt–chromium–molybdenum (CoCrMo) alloys, aluminum (Al) based ceramics and polyethylene are widely used for articulating components of total knee arthroplasty (TKA) and total hip arthroplasty (THA) implants. Titanium (Ti) alloys, such as titanium‐aluminum‐vanadium (TiAlV), are most often used as base materials for load‐bearing and stabilizing implant components.^[^ ^2^ ^]^ In revision arthroplasty, the elemental diversity is even more pronounced due to additional stabilizing components such as porous tantalum (Ta), which can compensate for insufficient bone integrity. Moreover, poly(methyl methacrylate) contains radiopacity enhancing zirconium dioxide (ZrO_2_) particles and is often used as orthopedic cement to fix implants in peri‐implant cancellous bone.

Despite the overall success of arthroplasty, local and systemic complications caused by release of metallic wear and corrosion products remain problematic.^[^ ^3^, ^4^ ^]^ Pairing CoCrMo alloys as articulating components of hip implants turned out to be unfavorable which is indicated by comparatively short primary implant survival strongly linked to the release of metallic nanoparticles and ions.^[^ ^5^, ^6^, ^7^, ^8^ ^]^ The characteristics of local adverse effects highly depend on the elemental composition and physicochemical properties of the released particles.^[^ ^9^ ^]^ Recently, it was shown that metal species are highly diverse in the peri‐implant membrane of metal‐on‐metal (MOM) hip implants.^[^ ^10^ ^]^ The diversity of metal speciation will even be more pronounced in the future due to recent advances in nanosized surface modifications of materials used for biomedical applications.^[^ ^11^ ^]^ While the ex vivo exposure assessment of metals released from implants with articulating metal surfaces is commonly described in literature, the steady release of metal wear debris from surfaces of load‐bearing but non‐articulating implant components is largely disregarded. This is particularly surprising, since histopathological examinations of the synovia‐like peri‐implant membrane reveal a comprehensive variety of metallic wear and corrosion products locally released from non‐articulating components of THA implants.^[^ ^12^ ^]^ Load‐bearing components have direct contact with adjacent cancellous bone structures and the bone marrow (BM) of patients at early post‐operative stages. During the post‐operative course, non‐biomimetic metal‐based materials induce a foreign body reaction, which results in encapsulation of the components by a collagen‐rich peri‐implant membrane with insulating properties.^[^ ^13^ ^]^ Local exposure has mostly been studied in the peri‐implant membrane and synovial fluid, but not comprehensively in the adjacent bone and BM.

Recent investigations suggest that the complex immune cell composition of the BM, and its capability to host adaptive immune cells, plays a crucial role in the development of metal exposure induced inflammation.^[^ ^14^ ^]^ A functional BM homeostasis is essential for human health, not only to constantly generate hematopoietic cells, but also for providing bone progenitor cells.^[^ ^15^, ^16^ ^]^ Unbalanced bone and BM tissue homeostasis is associated to various long‐term consequences like stress shielding induced periprosthetic osteopenia, periprosthetic fractures, osteolysis, and associated implant loosening.^[^ ^17^, ^18^, ^19^ ^]^ It is assumed that this is due to a local pro‐inflammatory environment resulting from particle exposure in the peri‐implant membrane which leads to increased osteoclastic and diminished osteoblastic differentiation and activity.^[^ ^20^, ^21^ ^]^ Recent ex vivo and in vitro studies provide evidence that metal exposure may directly affect osteoclasts and osteoblasts (OBs).^[^ ^22^, ^23^, ^24^ ^]^ Previous studies detected supraphysiological non‐particulate Co and Cr levels in the peri‐implant BM of patients with MoM articulation. These supraphysiological levels were associated with a loss of osteogenic capacity of human BM mesenchymal stromal cells (hMSCs), which implicated Co and Cr as being involved in the pathogenesis of peri‐implant bone loss.^[^ ^25^ ^]^ Yet, the toxicokinetics and toxicodynamics of the various metallic degradation products and their potential accumulation in peri‐implant bone and BM remain elusive.

The hypothesis of this work is that particulate as well as dissolved metals released from arthroplasty implants are not fully isolated by the peri‐implant membrane but are abundantly present in peri‐implant cancellous bone and intertrabecular BM. Using a unique synchrotron‐based X‐ray fluorescence (XRF) imaging setup, this study aims to qualitatively and quantitatively analyze the local toxicokinetics of released metallic wear and corrosion products and to identify their specific spatial distribution in bone and BM. Such an approach allows, for the first time, to analyze if metallic degradation products show a specific spatial distribution or uptake in human peri‐implant bone and BM on a micron‐ and nanoscale.

## 2 Results

### 2.1 Study Design and Experimental Strategy

**Figure 1:**
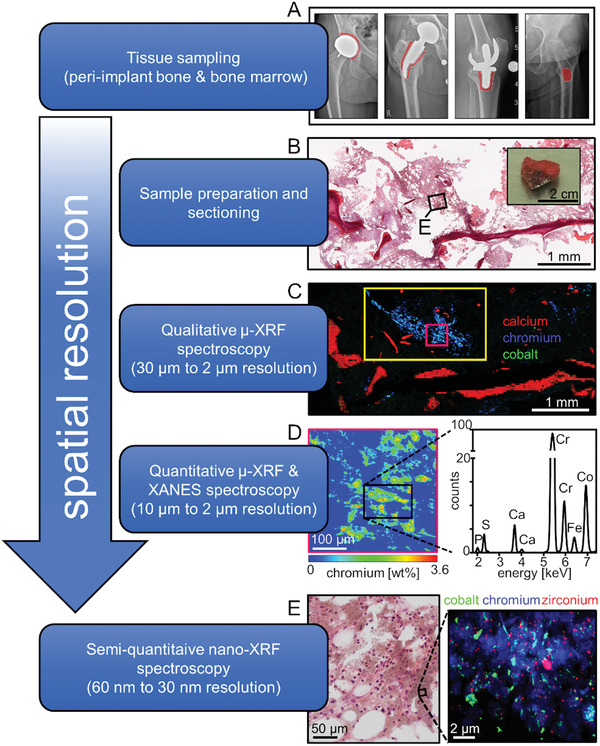
Representative experimental strategy for the spatially resolved multi‐metal exposure assessment by means of synchrotron micro‐ and nano‐XRF analyses of peri‐implant bone and BM. A) Cancellous bone including BM was intraoperatively harvested from close proximity to arthroplasty implants and from patient without implant as indicated by red areas in the depicted preoperative X‐ray radiographies. B) Representative image of a harvested specimen and H&E staining of peri‐implant cancellous bone including BM from a patient with hip resurfacing implant. C) Corresponding RGB (red: Ca, green: Co, blue: Cr) qualitative micro‐XRF maps in ascending order of resolution. Full image (30 µm spatial resolution), yellow rectangle (10 µm spatial resolution), pink rectangle (2 µm spatial resolution). D) Quantitative heat‐map of Cr weight fractions in the 2 µm resolved micro‐XRF map and an averaged XRF‐spectrum of marrow structures indicating predominant exposure to Co and Cr. E) RGB (red: Zr, green: Co, blue: Cr) qualitative nano‐XRF map with a spatial resolution of 60 nm indicating exposure to metal particles at various sizes and shapes.

In order to perform spatially resolved multi‐metal exposure assessment, 10 µm sections of peri‐implant cancellous bone including intertrabecular BM from 14 patients with different loosened knee and hip implants were analyzed by synchrotron micro‐ and nano‐XRF imaging. Corresponding specimens from six patients undergoing primary arthroplasty served as controls (**Table** **1**). Peri‐implant samples were intraoperatively harvested from close proximity to the implant and control samples were collected from the metaphyseal medullary cavity during primary implantation (**Figure** **1**A). For orientation during XRF‐mapping and for the definition of regions of interest (ROI), hematoxylin and eosin (H&E) stainings were performed on the sections adjacent to the sections used for XRF‐scans (Figure 1B). The general XRF‐scanning strategy was based on large fields of view micro‐XRF analyses, performed at the ID21 beamline with step size of 30 µm, for obtaining an overall picture of the metal distribution in cancellous bone and intertrabecular BM (Figure 1C). Thus, ROIs for mapping at higher resolution were defined from these overview maps and from histological evaluation. Criteria for ROI definition were focal metal exposure as well as the presence of biological alterations like osteolysis of cancellous bone, fibrous and inflammatory or necrotic changes within the marrow structures. Scans were performed over these ROIs at medium resolution (10, 3, and 2 µm) and quantitative mass fraction maps were calculated in whole scans or in specific areas of these maps (Figure 1D). Samples were then transferred to the nanoprobe ID16B. There, nano‐XRF mapping was performed on ROIs defined on medium‐resolution maps. For orientation purpose, an overview scan was performed with steps of 1 µm before scanning at resolutions of 60 and 30 nm (Figure 1E).

**Table 1:** Sample list, location of intraoperative sample extraction, and quantitative comparison of the individual samples regarding Co, Cr, and Ti content. Percentage of spatial mass fraction greater than background spatial mass fractions (= *Q*_0.999_ of all pixel intensities from control samples) and maximum spatial mass fractions of Co, Cr, and Ti. Data were obtained from all XRF‐scans with 2–10 µm spatial resolution

Sample ID	Revised implant	Sample location	*n* > *Q*_0.999_ [%]			Max value [wt%]		
			Co	Cr	Ti	Co	Cr	Ti
Control 1	‐ (native)	Femoral	0.31	0.12	0.02	0.04	0.47	0.11
Control 2	‐ (native)	Femoral	0.08	0.03	0.23	0.11	0.07	11.4
Control 3	‐ (native)	Femoral	0.19	0.01	0.00	0.02	0.01	0.05
Control 4	‐ (native)	Femoral	0.00	0.13	0.04	0.01	0.03	0.18
Control 5	‐ (native)	Femoral	0.00	0.28	0.05	0.02	0.51	0.20
Control 6	‐ (native)	Femoral	0.01	0.02	0.06	0.02	0.01	0.10
Case 1	Modular stem THA implant	Not classifiable	15.9	13.0	0.26	9.71	3.97	1.31
Case 2	Modular stem THA implant	Femoral	12.3	7.09	0.09	4.93	3.24	6.19
Case 3	Hip resurfacing implant	Acetabular	35.0	76.0	0.04	3.43	12.8	4.75
Case 4	Hip resurfacing implant	Not classifiable	25.4	49.9	0.01	1.80	6.19	0.18
Case 5	Hip resurfacing implant	Acetabular	2.85	98.2	0.04	0.89	2.17	0.55
Case 5	Hip resurfacing implant	Femoral	0.18	14.2	0.74	0.24	1.31	38.0
Case 6	Hip resurfacing implant	Acetabular	8.56	20.3	0.03	5.18	2.56	0.15
Case 6	Hip resurfacing implant	Femoral	0.23	0.61	0.94	0.55	1.04	0.36
Case 7	Primary THA implant	Acetabular	0.04	5.49	0.22	0.05	6.76	0.40
Case 8	Primary THA implant	Femoral	1.36	0.07	0.02	0.08	0.09	0.51
Case 9	Primary THA implant	Acetabular	0.14	1.60	9.21	1.50	0.53	48.4
Case 10	Primary THA implant	Femoral	1.63	1.47	2.00	2.82	1.17	32.9
Case 11	Multi‐component revision THA implant	Acetabular	0.44	0.70	0.25	0.24	1.41	5.53
Case 12	Multi‐component revision THA implant	Acetabular	0.79	0.44	4.78	0.49	2.15	30.9
Case 13	Primary TKA implant	Femoral	0.21	0.62	0.13	0.41	0.54	5.62
Case 14	Primary TKA implant	Tibial	41.5	59.2	0.00	3.61	4.12	0.29
Case 14	Primary TKA implant	Femoral	31.2	61.1	0.11	3.46	3.24	0.32

### 2.2 Identification of the Overall Exposure: Metal Mass Fractions in Analyzed Samples

For all samples analyzed, the underlying clinical case and secondary patient characteristics including implant data (Table S1, Supporting Information), prerevisonal radiographs (Figure S1, Supporting Information) and, if available in clinical records, local and systemic metal levels (Table S2, Supporting Information) are reported.

**Figure 2:**
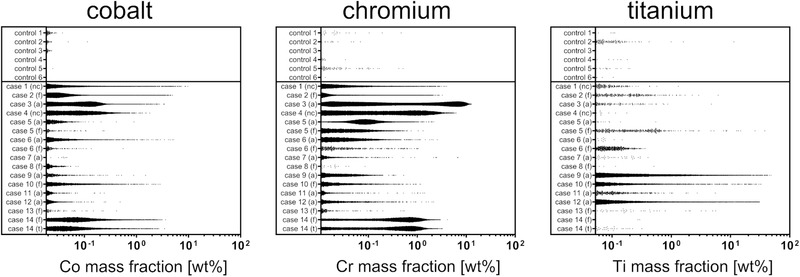
Distributions of the spatial mass fractions of Co, Cr, and Ti obtained from multi‐elemental XRF‐mapping. Depicted are all data points from maps with 2–10 µm spatial resolution which are greater than the metal specific 99.9% quantile of the overall data points obtained from the control samples (*Q*_0.999_ Co, 0.018 wt%; *Q*_0.999_ Cr, 0.011 wt%; *Q*_0.999_ Ti, 0.052 wt%). Data points from individual maps of the same samples were combined. Abbreviations: nc, sample location not classifiable; f, femoral; a, acetabular; t, tibial.

The nano‐ and micron‐resolved XRF‐analyses revealed that metals released from endoprostheses are abundantly present in acetabular, femoral, and tibial peri‐implant cancellous bone including BM with Co, Cr, and Ti particles of various sizes and shapes dominating the overall metal exposure. Supraphysiological metal quantities were identified in specimens from the proximity of various hip and knee endoprostheses independent of their specific design. Distinct regions within the samples contained metal mass fractions up to a double‐digit percentage. Other particulate metals like (zirconium) Zr and Ta were detected in individual cases. In order to benchmark the overall Co, Cr, and Ti contents of native (control) and peri‐implant samples, data points (mass fractions of each pixel) from scans with a spatial resolution of 2–10 µm were combined for each of the individual sample. All data points obtained from control samples were summarized to calculate the element‐specific upper 99.9% quantile (*Q*_0.999_). All data points below these upper *Q*_0.999_ of the control samples were defined as physiological background levels. Plotting of all data points revealed that supraphysiological Co, Cr, and Ti signals were highly abundant in peri‐implant samples of all implant types (**Figure** **2**). The relative amount of spatial mass fraction signals greater than *Q*_0.999_ as well as higher maximum spatial mass fractions also indicate supraphysiological quantities of these metals in peri‐implant samples compared to controls (Table 1). The distributions of the quantitative data points for Co, Cr, and Ti of all individual nano‐XRF maps with spatial resolution of 60 nm are provided in Figure S2, Supporting Information.

### 2.3 Specificity of the Spatial Distribution: Metal Exposure in Bone Marrow

**Figure 3:**
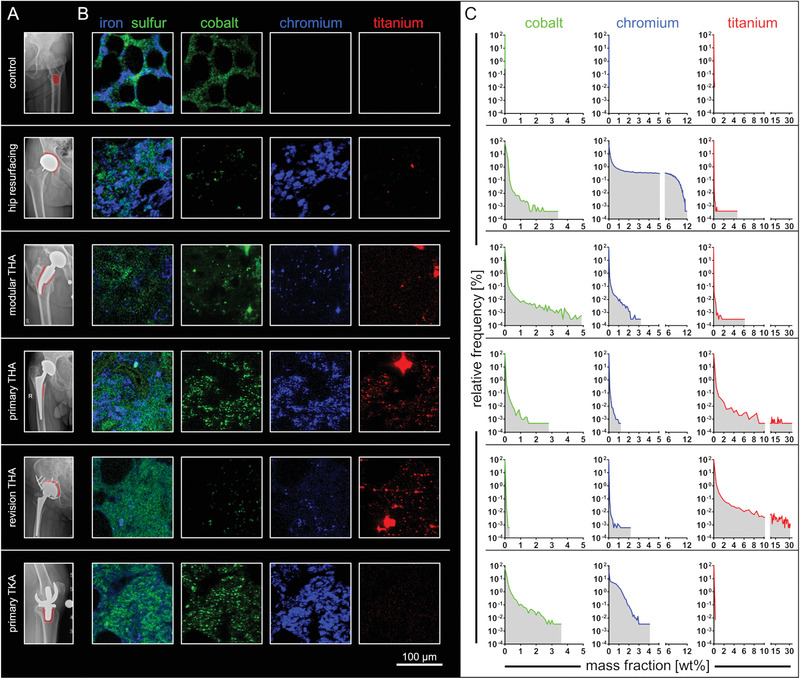
Co, Cr, and Ti were abundantly detected in peri‐implant bone marrow from various implant types and locations. A) Preoperative X‐ray radiographies. Red areas indicate the site of sample extraction. B) Qualitative RGB image sections of micro‐XRF maps with a resolution of 2 µm. Fe and S indicate matrix structures of the marrow. Mapping of Co, Cr, and Ti indicates the widespread dissemination of these metals in peri‐implant BM. Brightness and contrast of the RGB images were adjusted to represent the individual minimum and maximum amplitude of the signal. Scales are therefore not comparable from one sample to another. C) Frequency distributions of quantified mass fractions of each individual map and element indicate supraphysiological signals of Co, Cr, and Ti if compared to control (bin width [wt%]: Co, 0.1; Cr, 0.1; Ti, 0.5). Abbreviations: THA, total hip arthroplasty; TKA, total knee arthroplasty.

The overall multi‐elemental mass fraction analyses of peri‐implant specimens revealed substantial exposure to Co, Cr, and Ti (Table 1). To obtain an overall picture of the spatial frequency and quantity of metals in BM from the proximity of different implant setups, XRF‐maps with a resolution of 2 µm were analyzed with regard to spatial element distribution (**Figure** **3**). According to surgical reports, the cancellous bone samples including intertrabecular BM were intraoperatively collected in close proximity to the implant (Figure 3A) but distal to the peri‐implant membrane.

Qualitative iron (Fe) and sulfur (S) mapping indicates the presence of intertrabecular matrix and reveals variation regarding spatial distribution of these elements in the different samples. Qualitative mapping of the XRF‐scans from the control sample shows BM matrix‐specific physiological Co signals. Implant‐related metal exposure in peri‐implant BM samples was found to be dominated by Co, Cr, and Ti (Figure 3B). Quantitative frequency analyses of the Co, Cr, and Ti signals showed magnitudes higher mass fractions in the intertrabecular BM if compared to control (Figure 3C). Co and Cr were predominantly detected in peri‐implant intertrabecular BM from patients with hip resurfacing implant, TKA implant and modular THA implant. In contrast, detected Ti exposure was most dominant in patients with multi‐component revision THA implant and in femoral samples of patients with primary THA implant. In summary, peri‐implant BM is exposed to metals released from arthroplasty implants of various designs.

### 2.4 Characteristics of Cobalt and Chromium Exposure in Bone Marrow

XRF‐overview maps indicate that intertrabecular BM samples harvested in proximity to hip resurfacing implants and TKA implants are exposed to Co and Cr at the time of revision (Table 1). In‐depth analyses of these exposure scenarios were performed to resolve particle characteristics and locations of these two metals in the peri‐implant BM.

**Figure 4:**
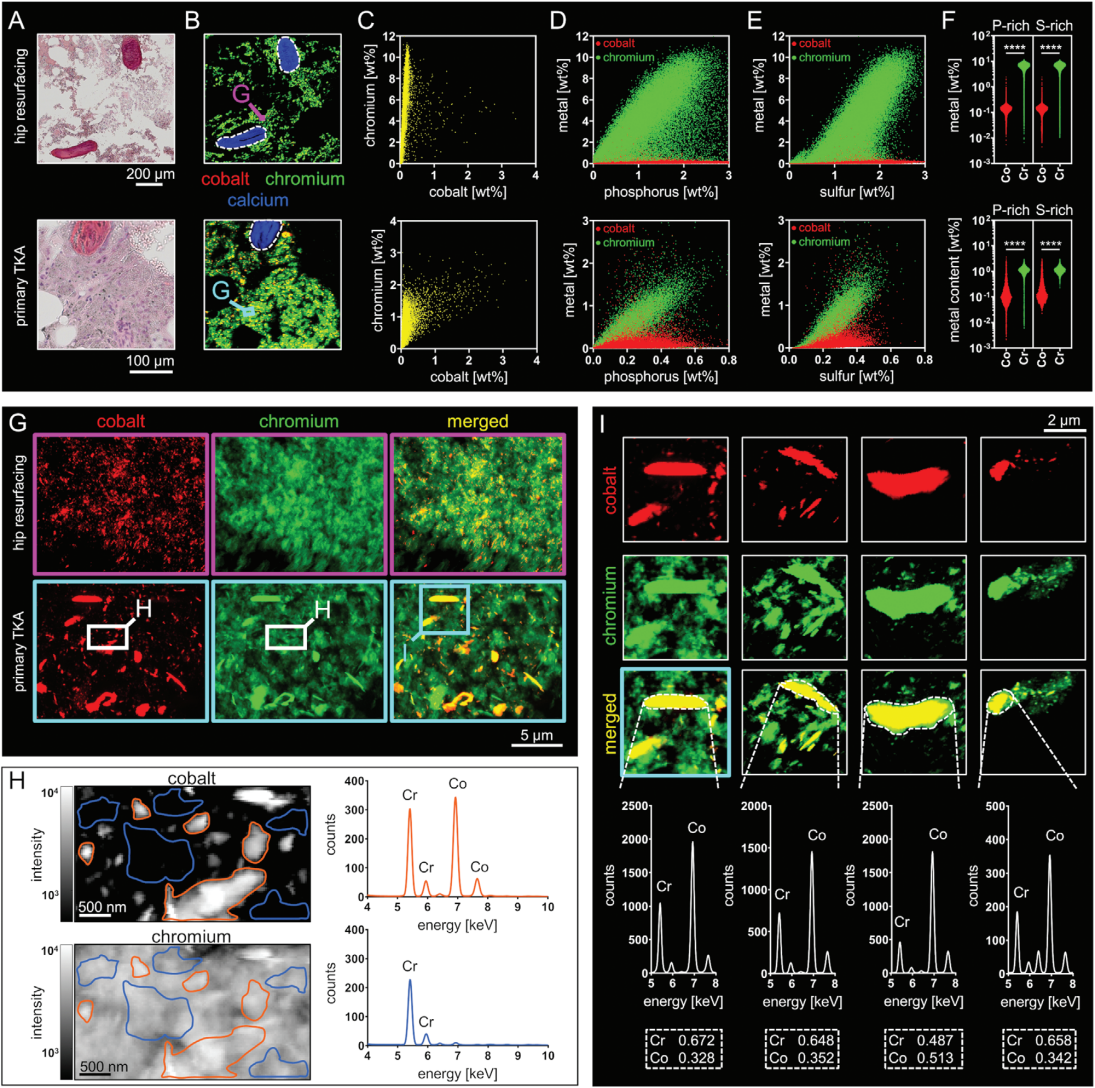
Non‐particulate Cr accumulates in BM matrix whereas Co was only found in the particulate state and co‐localized with Cr. A) H&E stainings of intertrabecular BM from the proximity of a hip resurfacing implant and a knee endoprostheses. B) Corresponding 2 µm resolved XRF‐maps of intertrabecular BM. The qualitative RGB imaging reveals spacious distribution of Cr and focal hotspots of Co co‐localized with Cr. C) Scatter dot plots of the Co and Cr special mass fractions (areas depicting cancellous bone were excluded, dashed lines) indicate that Co is exclusively co‐localized while Cr is also solitary detected in the BM. D) Scatter dot plots of the Co and P (red) and Cr and P (green) mass fractions indicate distinct co‐localization of Cr with P. E) Scatter dot plots of the Co and S (red) and Cr and S (green) mass fractions indicate distinct co‐localization of Cr with S. F) Cr versus Co mass fractions in P‐ and S‐rich data points by means of the upper 10% of data points regarding their S and P content. (Hip resurfacing: *n* = 22510 each group, primary TKA: *n* = 2853 each group; Mann–Whitney test (two‐sided); **** *p* < 0.001). G) Qualitative RGB imaging of nanoresolved (60 nm) XRF‐maps confirms the distinct deposition of Cr in the marrows’ matrix. Co and Cr containing particles of various sizes and shapes are abundantly represented in peri‐implant BM. H) Quantitative XRF‐heat maps at 30 nm resolution show non‐particulate Cr. Analyses of the according XRF‐spectra clearly demonstrate that the particles consist of both, Co and Cr, whereas areas of non‐particulate Cr do not contain Co. I) Quantification of Co and Cr contents of particles detected in the BM from a knee endoprosthesis (tibial, see (F)), knee endoprosthesis (femoral), hip resurfacing implant and a modular stem hip endoprosthesis (left to right) reveals lower Co:Cr ratios if compared to the bulk alloy. Stated numbers reflect the mass ratios of Co and Cr within the analyzed particles (dashed rectangles). Abbreviation: TKA, total knee arthroplasty.

To this end, regions of intertrabecular BM from the proximity of both implant types were chosen from histological stainings (**Figure** **4**A). Already at spatial micron resolutions (2 µm), it was found that Cr is highly abundant and homogenously distributed throughout the BM matrix structures, whereas Co was only focally detected (Figure 4B). Plotting of the spatial Co and Cr mass fractions obtained from the 2 µm resolved scans revealed that particles containing Co are exclusively co‐localized with Cr, whereas Cr was also detected without Co at high abundance (Figure 4C). To elucidate whether Cr accumulates within extracellular matrix or cellular structures of the marrow, the co‐localization of Cr with sulfur (S) and phosphorus (P) was investigated. S is a generally accepted indicator for extracellular matrix, while P is more specific for cellular structures. These low Z‐elements are detectable in the course of micro‐XRF analyses under vacuum conditions. Plotting of the spatial mass fractions indicated a distinct co‐localization of Cr with P (Figure 4D) and S (Figure 4E) within the analyzed peri‐implant BM of both implant types. Notably, Cr was exclusively detected co‐localized with S and P, indicating pronounced binding affinity of the released Cr to BM matrix and cellular structures. The Cr content within the P‐ and S‐rich data points are statistically significant higher if compared to the Co content in these data points (Figure 4F). This finding confirms the distinct co‐localization of Cr with P and S and indicates that Co does not manifest a local affinity for matrix binding. Since most particles released from arthroplasty implants are known to be significantly smaller than 2 µm, nanoresolved analyses were performed to confirm the metal‐specific distribution patterns. XRF‐scans at 60 nm resolution show substantial exposure to CoCr containing micron‐, submicron‐ and nanoparticles, confirm the distinct co‐localization of both metals and reveal dissolution clouds of Cr as characteristic distribution pattern of this metal (Figure 4G). These specific distribution patterns were observed in all Co and Cr exposed peri‐implant BM samples (Figure S3, Supporting Information). The homogenous distribution of Cr throughout the entire BM matrix becomes even more evident at a 30 nm resolution (Figure 4H). Cr was found to be present in the non‐particulate state and thus most likely to be covalently bound to matrix and cellular structures. The quantitative averaged XRF‐spectra of corresponding regions indicated that Cr, but not Co, is exclusively abundant in areas without submicron and nanoparticles. On the other hand, the detected particles exclusively contain both metals (Figure 4G,H). Nano‐XRF imaging of CoCr particles revealed Co and Cr containing particle cores and Cr containing particle coronas. Quantification of the Co and Cr content of particles in the peri‐implant BM from patients with different endoprostheses, revealed ratios that did not reflect the ratios of the bulk CoCrMo alloy (Figure 4I). In bulk CoCrMo alloy, the Co to Cr ratio is stated to be approximately 2:1, whereas the Co to Cr ratios in the analyzed particles were found to be 1:1 to 1:2. Taken together, more Co than Cr is released from peri‐impant particles, but Cr has a distinct binding affinity to BM matrix structures.

### 2.5 Exposure to Metals from Non‐Articulating Components

Beside the distinct exposure to Co and Cr, an unexpected finding of this study is the pronounced Ti exposure in all analyzed peri‐implant BM samples. The highest quantities of Ti particles were detected in the femoral surroundings of patients with THA implant and the acetabular surrounding of patients with revision THA implants (Figure 3). Even BM samples from the proximity of implants with relatively small Ti components, such as hip resurfacing implants or TKA implants, contain supraphysiological amounts of Ti (Table 1).

**Figure 5:**
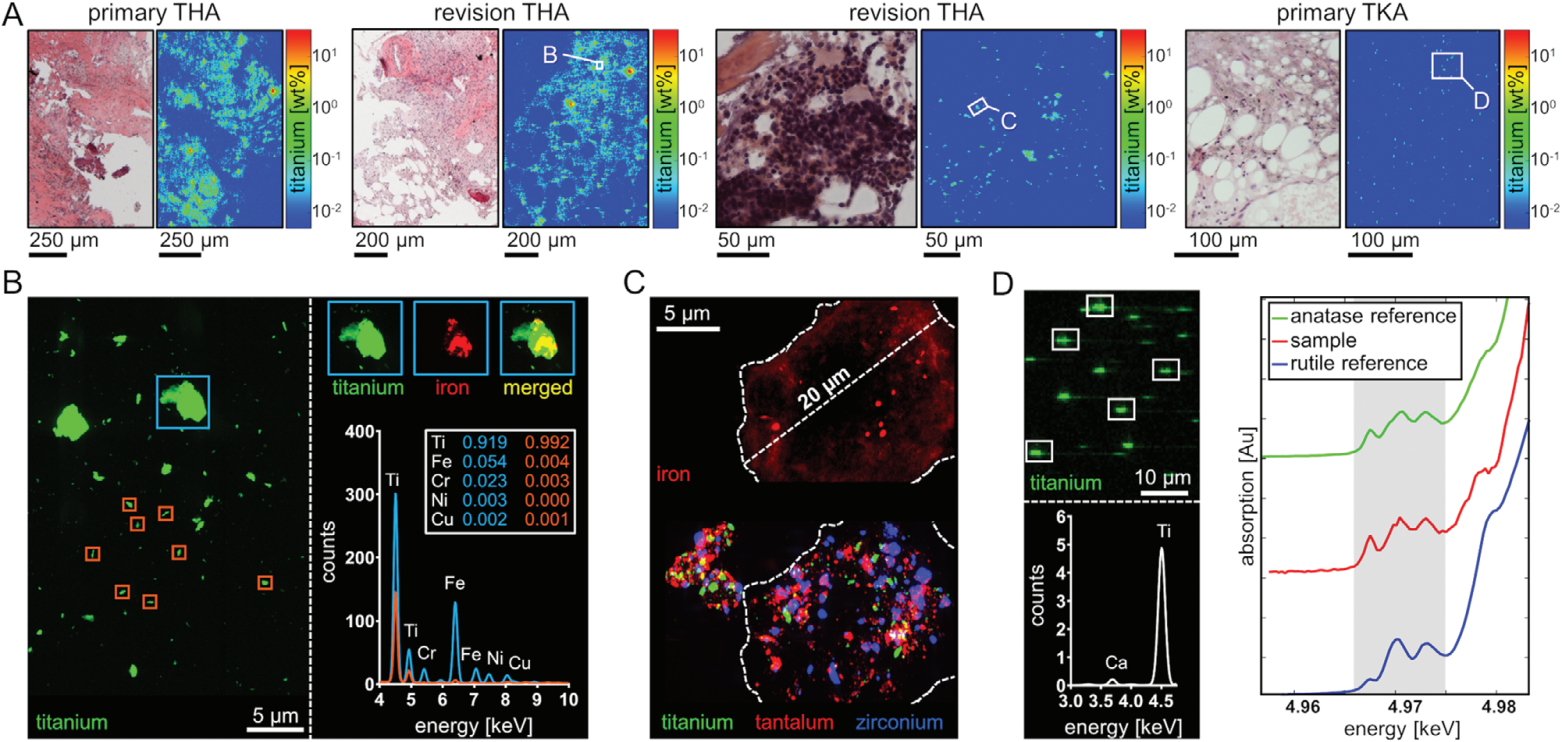
Exposure to particles from non‐articulating implant components in altered BM matrix. A) From left to right: Highly osteolytic bone remains and necrotic tissue from the femoral site of a patient with primary THA implant. Osteolytic bone and fibrotic marrow structures from a patient with failed revision THA implant. Highly osteolytic bone and inflammatory BM from a patient undergoing revision of a failed multi‐component revision THA implant. BM from the surrounding of a TKA implant is characterized by slightly fibrotic regions. 2 µm resolved XRF‐scanning and resulting heatmaps reveal extensive loads of Ti particles in necrotic and fibrotic zones. B) Nanoresolved analysis revealed micron‐ and nanosized Ti particles. Averaged XRF‐spectra reveal that Ti nanoparticles contain only traces of constituent alloy metals. C) Nanoresolved analysis of an inflammatory center reveals an enormous intra‐ and extracellular multi‐metal particle load. Size and shape of the depicted cell suggest the occurrence of a macrophage driven response. D) TiO_2_ particles within the BM from the tibial surrounding of a primary TKA implant. Qualitative imaging of submicron‐resolved (500 nm) XRF‐maps depict the presence of Ti within the analyzed sample section. Analysis of the averaged XRF‐spectra clearly demonstrates that the particles consist of Ti. Comparison of the resulting micro‐XANES absorption spectrum to previously measured spectra from TiO_2_ in the rutile and anatase phase clearly demonstrate a dominate anatase crystal proportion. Abbreviations: THA, total hip arthroplasty; TKA, total knee arthroplasty.

In the course of investigating the distribution, accumulation, and chemical speciation of Ti particles in these samples, it was noticed that the highest number of particles occur in tissue‐altered regions of the BM (**Figure** **5**A). These samples were characterized by highly osteolytic bone as well as fibrotic and necrotic BM. Extensive quantities of Ti micro‐, submicron‐ and nanoparticles were not only detected in fibrotic BM areas of the acetabular surrounding of a patient with multi‐component revision THA implant, but also in pronouncedly necrotic regions in proximity to a Ti stem of a THA implant (Figure 5A). In the latter case, nanoresolved XRF‐imaging revealed the presence of micron‐ and nanoscale Ti‐containing particles (Figure 5B). Averaged nano‐XRF spectra showed that micron‐sized Ti particles contain single digit percentage concentrations of alloy constituents such as iron and Cr. In contrast to the micron‐sized particles, Ti‐containing nanoparticles contain almost no traces of other metals (Figure 5B). Within necrotic tissue, nano‐ and micron‐sized particles were detected intra‐ and extracellularly in a focal inflammatory region with residual BM‐like structure (Figure 5C). In this sample from a patient who experienced a severe failure of a revision THA implant due to screw fracture and wear‐out of a cemented non‐articulating but load bearing trabecular Ta component, other metals than Ti like Zr and Ta were detected at high abundance. The identified cell within the inflammatory region fits the criteria for being a macrophage in terms of size and shape. It is generally accepted that chronic innate immune responses due to extensive particle load can lead to fibrotic and necrotic tissue changes.

Areas containing Ti particles were analyzed by micro X‐ray absorption near edge structure (micro‐XANES) spectroscopy since potential biological effects highly depend on the chemical and structural state of particles (Figure 5D). To this end, the peri‐implant BM of a patient undergoing revision of a TKA implant was analyzed by multi‐elemental XRF‐mapping to localize the Ti‐containing particles and to subsequently characterize the identified particles by micro‐XANES. The XANES spectra of the analyzed Ti particles (*n* = 5) show a characteristic which is consistent with spectra of titanium dioxide (TiO_2_). Crystal phases of TiO_2_ like the most frequent rutile and anatase, differ in their potency for inducing an inflammatory response. The comparison to rutile and anatase reference XANES spectra, clearly shows that the TiO_2_ dominantly occurs in the anatase crystal phase. A linear combination fitting of the generated spectra indicates that anatase is the predominant crystal phase of the analyzed TiO_2_ particles (Figure S2, Supporting Information). Collectively, the micron‐ and nano‐XRF analyses and micro‐XANES spectroscopy provide evidence for the release of metals, especially Ti, from non‐articulating implant components into the adjacent BM. In an inflammatory region, particles of multi‐metal composition can be found intracellular. Ti particles released from knee endoprostheses consist of TiO_2_ with dominant proportions of the comparatively pro‐inflammatory anatase crystal phase.

### 2.6 Metal Integration into Peri‐Implant Cancellous Bone

**Figure 6:**
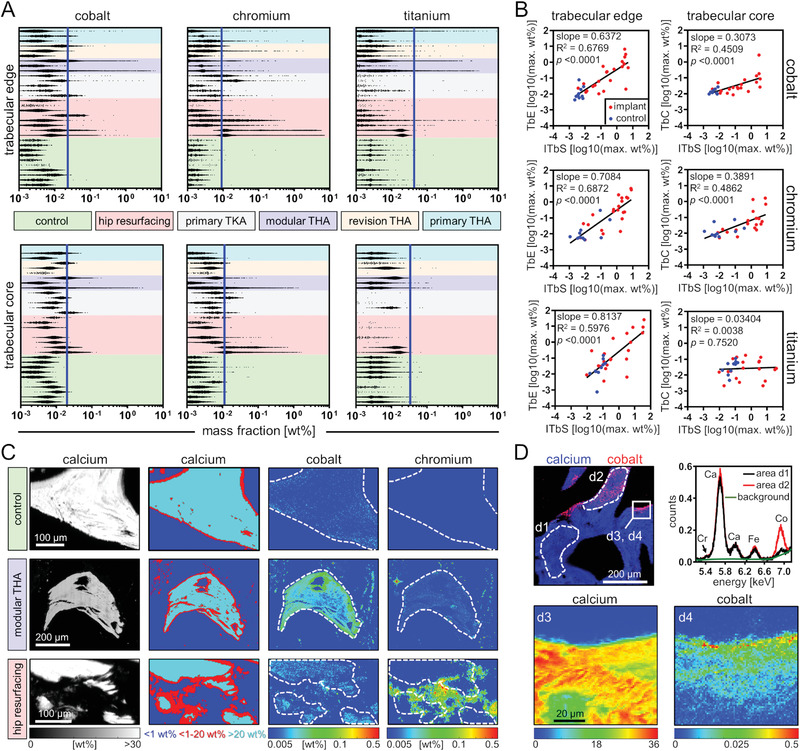
Co and Cr integrate into peri‐implant cancellous bone. A) The distributions of quantitative data points for Co, Cr, and Ti in trabecular edge regions (1–20 wt% Ca) and trabecular core regions (>20 wt% Ca) of micro‐XRF maps from peri‐implant cancellous bone samples. Blue lines indicate the upper 99.9% quantile of all control samples. B) Linear regression of maximum metal mass fractions. Two‐tailed Pearson correlation coefficient analyses (trabecular edge: Co, *n* = 32; Cr, *n* = 32; Ti, *n* = 32 and trabecular core: Co, *n* = 30; Cr, *n* = 30; Ti, *n* = 29). C) Quantitative heat maps of micro‐XRF scans (2 µm spatial resolution) of native cancellous bone, and peri‐implant cancellous bone samples. From left to right: quantitative Ca maps, spatial view of thresholding of the different regions regarding Ca content, quantitative Co and Cr maps. D) Co integration into intact peri‐implant cancellous bone. Top left to bottom right: qualitative image of a micro‐XRF map (5 µm spatial resolution), averaged micro‐XRF spectra of a region indicative of low Co content (d1) and high Co content (d2), quantitative Ca (d3), and Co (d4) heatmaps of micro‐XRF scans (1 µm spatial resolution) indicates gradual Co integration. Abbreviations: TbE, trabecular edge; TbC, trabecular core; iTbS, intertrabecular space; THA, total hip arthroplasty; TKA, total knee arthroplasty.

Since the obtained analyses revealed significant metal exposure within intertrabecular BM, it was subsequently investigated whether and to what extend metals integrate into cancellous bone. Maps with 2 and 10 µm spatial resolution containing bone trabeculae were analyzed (**Figure** **6**). Threshold levels of the calcium (Ca) content were defined for assigning the individual data points to trabecular bone. Data points with a Ca content greater than 1 wt% and less than 20 wt% were defined as trabecular edge (TbE) regions. Data points with a Ca content greater than 20 wt% were defined as trabecular core (TbC) regions.

Concentrations of Co, Cr, and Ti were found to be several orders of magnitude higher in peri‐implant TbE regions than in control samples. While Co and Cr were abundantly present in TbE regions of all peri‐implant samples, Ti exposure in peri‐implant TbE regions was dependent on the implant type and was generally less pronounced and especially low in samples obtained from TKA implants. In TbC regions, no significant exposure to Ti was detected, whereas Co and Cr also integrated into peri‐implant TbC regions, particularly into peri‐implant trabeculae of hip resurfacing implants and modular THA implants (Figure 6A). The integration of Co and Cr into peri‐implant trabeculae was further confirmed by regression analyses of metal contents in the intertrabecular space (iTbS) and in the trabeculae. This analysis revealed a statistically significant positive correlation between maximum mass fractions of Co and Cr in the iTbS and both regions (TbE and TbC) of the trabeculae. Accordingly, no correlation was found between the maximum mass fractions of Ti in iTbS and TbC regions, confirming that Ti is not integrated into intact peri‐implant trabeculae (Figure 6B). Analyses of spatially resolved quantitative heat maps of trabeculae from the proximity of a hip resurfacing implant and a modular THA implant indicate that Cr is rather focally present, particularly in TbE regions, and not consistently distributed in TbC regions. In contrast, a uniform distribution of Co in peri‐implant trabeculae indicates its pronounced integration affinity (Figure 6C). Spatially resolved maps of intact femoral bone trabeculae in close proximity to a THA implant illustrate the onset of Co integration, which is characterized by gradual integration from TbE regions to TbC regions (Figure 6D). Taken together, the BM and cancellous bone analyses show that Co and Cr integrate into peri‐implant cancellous bone and their maximum concentrations strongly correlate with those found in intertrabecular BM. In contrast, even in cases of very pronounced Ti exposure within intertrabecular BM, there is no Ti integration into the trabecular core.

### 2.7 Validation of the Metal‐Specific Distribution: Bone/Bone Marrow‐on‐a‐Chip

**Figure 7:**
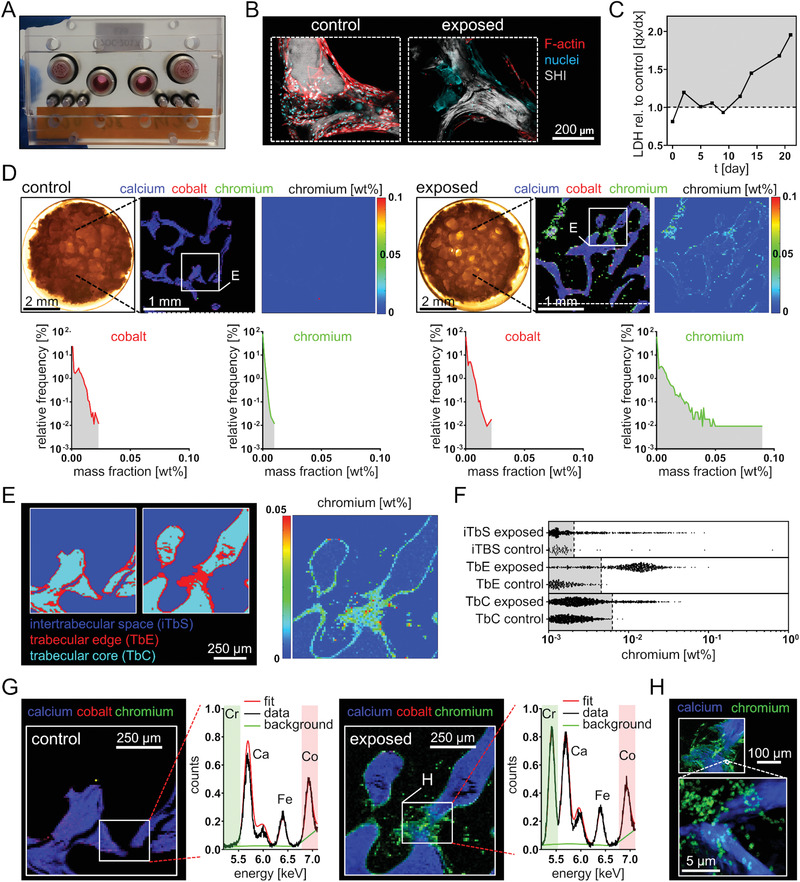
Exposure simulation in a bone/BM‐on‐a‐chip system revealed Cr accumulation in bone trabeculae and intertrabecular matrix. A) Microfluidic chip system with repopulated human cancellous bone. B) Confocal fluorescence (F‐actin, nuclei) and second harmonic imaging (type I collagen) of non‐exposed cancellous bone indicates bone lining cells and non‐intact cells after Co and Cr exposure (2.5 mg L^−1^ each). Z‐stack: control, 105 µm; exposed 150 µm. C) Lactate dehydrogenase determination (LDH) revealed an increasing LDH release over the experimental period after Co and Cr exposure. LDH values are depicted as relative values to the LDH values from the non‐exposed chip of the corresponding time point. D) Light microscopy images of the exposed and non‐exposed scaffold at day 18 of metal exposure point to matrix presence in the intertrabecular space. Qualitative RGB images of 30 µm resolved XRF‐maps indicate Cr accumulation in the scaffold after exposure to Co and Cr. Quantitative heatmap of Cr signals and frequency distributions prove the Cr accumulation. E) Spatial view (10 µm resolution) of the thresholding of different regions regarding calcium content. F) Cr signals and distributions of the quantitative data points indicate distinct Cr accumulation in the intertrabecular matrix and bone trabeculae. Gray areas indicate region‐specific background Cr levels which were defined as all signals below the upper 99.9% quantile (dashed lines) of the control sample. G) Averaged XRF‐spectra indicate Co background in both scaffolds and prove the Cr accumulation. H) Qualitative RGB images of different regions at 2 µm (top image) and 60 nm resolution (bottom image) indicate Cr exposure in bone and bone marrow matrix. Abbreviations: SHI, second harmonic imaging; LDH, lactate dehydrogenase TbE, trabecular edge; TbC, trabecular core; iTbS, intertrabecular space.

Multi‐metal XRF‐analyses showed that Co and Cr integrate into peri‐implant cancellous bone in a non‐particulate state and that Cr exhibits a high binding affinity for BM matrix. To further investigate this distribution pattern, a bone/BM‐on‐a‐chip system (**Figure** **7**A) was used to model in vivo exposure to dissolved (non‐particulate) Co and Cr in vitro, in order to validate their integration into cancellous bone and BM matrix also after short‐term (subacute) exposure. Bone/BM‐on‐a‐chip cultures were exposed to divalent Co and trivalent Cr ions at a concentration each of 2.5 mg L^−1^ in the microfluidic system for 20 days. Corresponding non‐exposed bone/BM‐on‐a‐chip cultures served as controls.

Confocal fluorescence imaging of in vitro cultured scaffolds revealed an intact cellular layer on the trabecular surface in the absence of Co and Cr ions and diffuse DNA deposits and non‐intact actin cytoskeletons following exposure to Co and Cr ions (Figure 7B). Lactate dehydrogenase (LDH) release was used as a surrogate marker for cytotoxicity and revealed an elevated LDH release in cell media supernatants of the exposed bone/BM‐on‐a‐chip culture over the culture period (Figure 7C). The increased LDH concentration together with the observed cell morphological changes seen by 3D imaging of trabeculae are indicative of cytotoxic effects after subacute exposure to soluble Co and Cr.

Overview micro‐XRF mapping at a spatial resolution of 30 µm and the distribution of the signal intensity frequency clearly indicated higher Cr signals in exposed culture compared to the control (Figure 7D). In contrast, a higher Co signal was not detected in the bone trabeculae nor in the intertrabecular space of the exposed culture. Micro‐XRF analyses at higher resolution, showed that Cr accumulates in the intertrabecular matrix (Figure 7E). Thresholding, equal to the ex vivo analyses revealed exposure in the intertrabecular space, in bone trabeculae and most pronounced at the trabecular edge (Figure 7F). A matrix accumulation or bone integration of Co was not observed at higher resolution. Average XRF‐spectra confirmed Co background in both scaffolds which is comparable to the background found in the ex vivo control samples and prove the Cr accumulation (Figure 7G). Micro‐XRF mapping (Figure 7G) and high‐resolution nano‐XRF mapping (Figure 7H) confirmed the in vivo finding that Cr has a high bone and BM matrix binding affinity. In summary, the bone/BM‐on‐a‐chip culture shows that exposure to dissolved Co and Cr at clinically relevant concentrations leads to direct cytotoxic effects and confirms the integration of Cr into cancellous bone and binding to intertrabecular matrix.

## 3 Discussion

Particulate and non‐particulate metals released from endoprostheses are abundantly represented in peri‐implant cancellous bone and intertrabecular BM. The spatially resolved elemental analyses, performed in this study, in the micron‐ and nanorange, provide unique insights regarding concentration, distribution, location, and accumulation of metallic degradation products in peri‐implant bone and BM. These results shed new light on the local toxicokinetics of wear and corrosion products and highlight the urgent need for bone and BM‐specific risk benefit evaluation of implant materials.

Supraphysiological amounts of Co and Cr were detected in the peri‐implant BM of patients with hip resurfacing implant or TKA implant. It is well known that hip resurfacing implants are predisposed to local release and systemic distribution of high Co and Cr quantities.^[^ ^26^ ^]^ The pronounced Co and Cr exposure in the BM per se, exposure in the peri‐implant BM of patients with knee endoprostheses and the distinct differences between Co and Cr regarding the local kinetics are novel findings. Cr was found in the particulate state in the micron‐ and nanoscale. Interestingly, in the particulate state Cr was found to be exclusively co‐localized with Co and nanoresolved images of micron‐sized particles depict Cr‐rich coronas of these particles pointing on a release of Cr from the edges of these particles. This finding is in line with previous elemental analyses of the chemical speciation of CoCr particles within the peri‐implant membrane detecting Cr(III) salts and oxides to surround the particles.^[^ ^27^ ^]^ In the BM, Cr distinctively accumulates within reticular and cellular structures. The high amounts of non‐particulate Cr point to binding affinity to the local connective tissue. The pronounced co‐localization of non‐particulate Cr with phosphorus and sulfur suggests its binding to anionic structures of matrix molecules. Possible binding partners are glycosaminoglycans with polyanionic side chains such as chondroitin sulfate and keratan sulfate, which are abundantly represented in the connective tissue of the BM.^[^ ^28^ ^]^ The ability of Cr(III) to bind collagen‐rich connective tissue structures and thereby denature and preserve extracellular matrix by cross‐linking has been used for centuries in chrome tanning of leather. This mechanisms could be assumed to occur when comparing the results presented here with a recent study on Cr binding in leather.^[^ ^29^ ^]^ Moreover, Cr(III) is capable of covalent interactions with cellular macromolecules such as extracellular phospholipids.^[^ ^30^ ^]^ The specific cellular or extracellular biomolecules to which Cr binds to in the BM cannot be conclusively answered by XRF‐analyses. However, BM structures seem to be prone to Cr binding since classical toxicokinetic studies in rodents show the most significant retention of Cr in this tissue.^[^ ^31^ ^]^ Cr(III) phosphate has been shown to be the most commonly detected corrosion product in the peri‐implant membrane.^[^ ^32^ ^]^ In the future, XANES analyses could potentially clarify the additional presence of Cr(III) orthophosphate or other inorganic Cr conformations in peri‐implant BM. The distinct binding affinity of Cr(III) was additionally proven by subacute exposure in a bone and BM on‐a‐chip system. A clinically relevant Cr concentration of 2.5 mg L^−1 [^ ^25^ ^]^ led to Cr accumulation in the intertrabecular matrix. The observed quantities within the matrix after 20 days of exposure were approximately two magnitudes lower if compared to the ex vivo analyses. This leads to the possible implication that a constant replenishment of Cr ions from particle deposits leads to summation effects in vivo.

Beside Cr, Co was also detected in peri‐implant BM at high abundance. U.S. and European authorities listed Co compounds that release Co ions in vivo as potential human carcinogens.^[^ ^33^, ^34^ ^]^ Since Co ions are classified as potential carcinogens and supraphysiological quantities of Co could be detected in BM of patients with endoprostheses, another implication of this work is that Co exposure in the BM should also be considered when assessing a potential cancer risk. However, existing literature on the risk of cancer development following implantation of hip endoprostheses with MoM pairing is controversial. A current implant registry studie clearly indicate no evidence for an overall higher cancer risk in patients with MoM pairings if compared to patients with non‐MoM pairings.^[^ ^35^ ^]^ Generally, a long time‐period from tumor initiation to progression must be considered in many cancers and the advanced age of the arthroplasty clientele may mask potential exposure‐related effects. Furthermore, none of the available epidemiological studies on a potential cancer risk related to metallic endoprostheses, has been controlled for the actual exposure, that is, for systemic and local metal levels.

The main difference in the local kinetics of Co compared to Cr is that Co was exclusively detected in the particulate state (always co‐localized with Cr) in matrix structures of the BM. This particle characteristic is in line with results from a study investigating the elemental composition of wear particles in the peri‐implant membrane of patients with MoM pairings.^[^ ^27^ ^]^ Interestingly, in the case of metal release, the ratio of systemic to local levels is higher for Co than for Cr.^[^ ^26^ ^]^ Reasons for this are most likely the higher dissolution capacity of Co, its concomitant lower binding affinity to matrix molecules and also its distinct binding affinity to soluble transport proteins as albumins and hemoglobin, which are highly abundant in the BM.^[^ ^36^, ^37^ ^]^ In contrast, Cr forms deposits in the local matrix, which are released over a prolonged time period, whereas Co is characterized by a higher potential for systemic distribution but also for excretion.^[^ ^38^ ^]^ These kinetics explain why, in cases of severe failure of MoM endoprostheses and local metal release, systemic Co versus Cr levels increase more but also decrease faster following revision surgery.^[^ ^39^, ^40^ ^]^ In addition, Co passively dissolves more rapidly in comparison to Cr. Nanoresolved XRF‐maps of micron‐sized CoCr particles indicate that the Co to Cr mass ratio is altered toward a lower Co content if compared to bulk CoCrMo alloy.

Notably, fractions of the locally released Co, Cr, and Ti integrate into the trabecular edge regions of peri‐implant cancellous bone. Co and Cr are able to further integrate into trabecular core regions especially in proximity of modular stem hip endoprostheses, while only Co was found to equally distribute within the trabecular core of bone from the surrounding of different types of endoprostheses. Classical studies on the toxicokinetics of Co support our findings regarding Co integration into bone. It has been shown that Co integrates into trabecular and cortical surfaces.^[^ ^41^ ^]^ Studies investigating the Co and Cr content in mineralized matrix in femoral heads collected at revision surgery of hip resurfacing implants identified an increase of the Co content in peri‐implant bone and a correlation of the Co content with implant life time.^[^ ^42^, ^43^ ^]^ This is interesting since these hip resurfacing implants are known to cause osteolytic lesions at higher prevalence if compared to other hip implant designs.^[^ ^5^, ^44^ ^]^ Moreover, there is evidence that dissolved Co interacts with type I collagen, the principle protein in bone, by binding to collagen fibrils leading to diminished collagen density.^[^ ^45^ ^]^ In contrast, in vitro exposure to Co ions in a 3D bone/BM‐on‐a‐chip system did not lead to enhanced Co integration into bone trabeculae. This indicates that Co integration into a native bone matrix does not occur passively and requires active bone remodeling over a longer time period than in the performed in vitro setting. The identified regions of ex vivo peri‐implant trabeculae with distinct Co integration allow for the implication that osteoblasts (OBs), osteocytes, and osteoclasts are directly exposed to Co. This adds weight to the clinical relevance of many studies investigating the osteogenic and inflammation‐inducing potential of osteoblastic cells following Co exposure in vitro and the derived implication on its direct involvement in the pathomechanism of osteolysis.^[^ ^46^, ^47^, ^48^ ^]^

Furthermore, it must be considered that not only Co and Cr account for the overall metal exposure in peri‐implant BM. Particularly in the adjacent BM of stemmed prostheses and multi‐component revision implants, large quantities of particles consisting of Ti, Ta, and Zr were detected both intracellularly and extracellularly in the micron‐, submicron‐ and nanorange. Primarily, these metals were detected in fibrotic and necrotic regions of the peri‐implant BM. Necrotic and fibrotic alterations of tissues are commonly late changes that occur following chronic inflammation. The large quantities of Ti particles in these conditions suggest that Ti particles are persistent in the BM tissue structures over a long period of time. Particles consisting of Ti, Ta, and Zr and their oxides are characterized by high persistency due to low solubility in vivo and are known to induce chronic inflammation, which is characterized by a non‐specific macrophage‐dominated immune response.^[^ ^49^, ^50^, ^51^ ^]^ Depending on particle size, this innate response leads to foreign body reactions or phagocytosis by macrophages.^[^ ^52^ ^]^ Both processes lead to macrophage activation, which consequently induces a pro‐inflammatory milieu and thereby paracrine cellular effects. Proinflammation in proximity to bone is associated with the clinical problem of sterile peri‐implant osteolysis and attributed to reduced osteoblastogenesis and increased osteoclastogenesis.^[^ ^20^ ^]^ Other important biological consequences of pronounced exposure to stable particles and concomitant chronic inflammation in the peri‐implant membrane are known to be fibrosis and necrosis.^[^ ^53^ ^]^ A histopathological pattern which was also observable in peri‐implant BM samples analyzed in this study. Biological consequences of exposure to Ti‐containing particles should be systematically analyzed in vitro and ex vivo at higher sample sizes.

In the course of assessing potential risks associated with Ti‐containing particles released from non‐articulating components, it should be noted that exposure‐related biological consequences not only depend on particle size and solubility but also on particle morphology and the crystal phase of metal oxides.^[^ ^54^ ^]^ The unexpected result of pronounced exposure to Ti‐containing particles in BM of all mapped peri‐implant BM specimens led us to thoroughly characterize these particles. The analysis of the chemical and structural state of Ti‐containing particles revealed that TiO_2_ in the anatase crystal phase dominates over the rutile phase. Generally, like many metals, Ti and Ti alloys, as used for orthopedic implants, form an oxide passivation layer on the metal surface. The ratios of the different TiO_2_ crystal conformations thereby depends on pressure, temperature, and chemical environment during the formation of the passivation layer.^[^ ^55^, ^56^ ^]^ High levels of anatase on the surface of implant materials are likely intentional because in vitro and in vivo studies have shown anatase layers to be beneficial for osseointegration of the material.^[^ ^57^, ^58^ ^]^ However, in vitro and in vivo studies focusing on the risk assessment of nanoparticulate TiO_2_ showed that exposure to TiO_2_ in the anatase state leads to more pronounced inflammatory, cytotoxic and genotoxic effects when compared to TiO_2_ particles in other crystal phases.^[^ ^59^, ^60^, ^61^ ^]^ Such effects are the basis for currently ongoing controversial discussions about the reclassification of TiO_2_ particles by European regulatory authorities. Due to the harmful effects of TiO_2_ particles and to the detection of TiO_2_ particles in peri‐implant BM in our study, we believe in the future it will be necessary to systematically analyze Ti‐based bulk materials that are currently used in orthopedic implants with respect to their surface characteristics. Additionally, in depth characterization of in vivo released particles and their ability to distribute in the human body is necessary in order to evaluate the Ti exposure‐related risks to patients.

Our results show that supraphysiological quantities of metals are found in all peri‐implant bone and BM samples. Co, Cr, and Ti dominate the metal exposure in peri‐implant BM where the overall elemental distribution is comparable to that found in the peri‐implant membrane.^[^ ^62^ ^]^ These results suggest that the peri‐implant membrane is permeable to the products of metallic wear and corrosion and implies BM and cancellous bone as future target organs in preclinical testing of implant materials. Preclinical cytotoxicity testing in the course of regulatory approval has so far been carried out mainly by using immortalized cell lines in 2D cultures (ISO 10993–5). Possible future regulatory extensions or replacements should include the integration of in vitro models that better emulate the cellular diversity of the BM.^[^ ^63^ ^]^ Moreover, in vivo biocompatibility tests of arthroplasty materials should be performed at the actual site of implantation in order to enhance the validity of the results compared to subcutaneous models. Pathological changes in the peri‐implant bone have recently been added to the framework of the histopathological consensus classification.^[^ ^64^ ^]^ However, a histological classification for diagnostics of peri‐implant BM pathologies is pending. Toxic‐ or allergy‐induced hypersensitivity reactions have only been described for the peri‐implant membrane. In case of local exposure, these specific immune responses might also occur beyond the membrane, since the immune cell compartment of the BM is complex and known to be a homing site for antigen specific cells.^[^ ^65^ ^]^ Effector memory T cells are key players in delayed type hypersensitivity toward hapten‐carrier adducts formed by metal ions like Co und Ni.^[^ ^66^ ^]^ Preclinical models, as presented in this study, are required to emulate the full cell and matrix complexity of peri‐implant bone and BM to provide a microenvironment closer to that found in vivo.

### 3.1 Limitations of the Study

The data presented here indicate exposure to metals within bone and BM which were released from various types of endoprostheses. Due to the complexity of the methods involved, we were only able to measure relatively few samples resulting in a small sample size. Additionally a small intra‐operatively harvested specimen only represents a small part of the overall local exposure, which does neither allow for comparing exposure‐related biological alterations between specimens nor for comparing general clinical advantages versus disadvantages of the analyzed implants with other implant types. Furthermore, the detected exposure levels only represent a retrospective snapshot at the time of implant revision, we cannot therefore draw any conclusions about the clinical course prior to revision surgeries. However, in‐depth analyses of individual exposure scenarios indicate pronounced metal‐specific accumulation in peri‐implant bone and BM and a general similarity between different implant types regarding local kinetics of Co, Cr, and metals released from non‐articulating components. We were able to confirm the Cr‐specific accumulation within intertrabecular BM matrix and bone trabeculae in vitro by a 3D bone/BM‐on‐a‐chip system. However, for a future validation of this in vitro system as a potential preclinical test system, it is necessary to confirm this finding with cells and particles from individual donors at various exposure levels and durations to emulate the full range of potential variability.

## 4 Conclusion

The obtained data demonstrate that metals released from various arthroplasty implants lead to multi‐elemental exposure in surrounding bone and BM. In summary, Cr distinctively accumulates in matrix structures of peri‐implant BM, Co and Cr integrate into the peri‐implant cancellous bone and Ti particles were predominantly found in peri‐implant regions of the BM affected by fibrotic and inflammatory changes.

The rather low oxidative stability of the CoCrMo alloy compared to other implant materials and associated concerns have led to the development and launch of many alternative implant materials in recent years. In the field of THA, CoCrMo‐free implants are becoming increasingly important. Long‐term data from implant registries confirm satisfactory results of CoCrMo‐free THA.^[^ ^67^ ^]^ In TKA, CoCrMo‐free implants and corresponding long‐term data are not yet available to the same extent as in THA.

Despite problems regarding local metal dissolution and systemic distribution of Co and Cr ions, local persistency of more stable debris, that is, consisting of TiO_2_, must also be considered. We are convinced that risk‐benefit evaluation of medical devices should not only comprise biocompatibility testing of bulk materials but also of wear and corrosion products. The findings obtained in this study help to define clinically relevant concentrations and metal specifications and thus to perform more realistic in vitro testing in the future. There is a distinct need for advanced approaches that facilitate reliable preclinical testing using cells or engineered tissues that emulate the peri‐implant biology to further risk benefit evaluation of current and future implant materials. This would support keeping implant safety at the highest possible level.

## 5 Experimental Section

Detailed information on chemicals, buffers, and equipment used in the course of sample harvesting and preparation are listed in Table S3, Supporting Information.

##### Patient Recruitment and Sample Harvest

Peri‐implant cancellous bone specimens including BM were collected from 14 patients undergoing aseptic revision surgery of primary or revision arthroplasty implants of the hip or knee joint. In addition, native non‐exposed samples were collected from seven patients undergoing primary implantation of artificial hip and knee joints. Six samples served as controls for XRF‐imaging. Primary cells for the bone and BM‐on‐a‐chip culture were isolated from one sample. The study was approved by the Institutional Review Board (IRB) of the Charité – Universitätsmedizin Berlin (IRB approval EA1/194/13). All patients gave written informed consent.

##### Sample Preparation and Sectioning

After intraoperative sample harvesting, the peri‐implant samples were fixed, embedded and sectioned prior to XRF‐mapping. In brief, the cancellous bone specimens with an approximate diameter of 0.5–2 cm were directly placed into 4% paraformaldehyde solution. Following overnight fixation, the specimens were washed with phosphate‐buffered saline and placed into embedding cassettes. For dehydrating the specimens, an ascending ethanol gradient (70%, 80%, 96%, and 100%) was performed for 24 h in each step. After completing dehydration by incubation with xylene for 3 h, the specimens were preinfiltrated in methyl methacrylate, which contains 0.5% m/v benzoyl peroxide / dicyclohexyl phthalate, by applying a partial vacuum at 400 mbar for 10 min and then incubation at normal pressure at 4 °C for 3 days. In addition to the preinfiltrated step, a subsequent 7‐day infiltration step was performed with additional supplementation of 8% m/v poly(methyl methacrylate). Final embedding of the specimens was performed in a polymerization solution consisting of nine parts methyl methacrylate containing 16% m/v poly(methyl methacrylate) plus 0.6% m/v benzoyl peroxide / dicyclohexyl phthalate and one part methyl methacrylate containing 8% v/v 3,5,*N*,*N*‐tetramethylaniline and 4% v/v 1‐decanethiol. For polymerization, a partial vacuum of 400 mbar was applied for 10 min before the specimens were incubated for at least 24 h at 4 °C. The polymer blocks were sectioned at a rotary microtome with a tungsten carbide blade under constant moistening by 30% ethanol. 10 µm thick sections were prepared for the XRF‐analyses and 7 µm thick section for histological staining. XRF‐sections were placed between two pieces of a 4 µm thin ultralene foil and dried overnight at room temperature. Sections for histological staining were glazed with a 20% 2‐methoxyethyl acetate in 70% ethanol solution, covered with polyethylene foil and dried at 60 °C for 48 h under constant compression.

##### Histological Staining and Imaging

H&E stainings on separate but adjacent sections were performed to achieve optimal orientation during XRF‐scanning. Standard protocols for plastics removal by 2‐methoxyethyl acetate and subsequent H&E staining were performed.^[^ ^68^ ^]^ Light microscopy was performed on a Zeiss Axio Scope. All pictures were taken with the use of a Zeiss Axiocam MRc microscope camera.

##### Micro‐X‐Ray Fluorescence Mapping

Multi‐elemental XRF‐mapping in the micron scale with a tunable monochromatic energy above the Co K‐edge was performed at beamline ID21 of the European Synchrotron Radiation Facility (ESRF). The experimental setup of this end‐station was adapted as previously described.^[^ ^69^ ^]^ The ultralen microfoil covered sections were mounted in custom‐made sample holders. An excitation energy of 7.8 keV was set up to detect the elements Al, P, S, Ca, Cr, Ti, V, Mn, Fe, and Co. Undulators harmonics were removed using a double mirror with Ni coating. High monochromaticity was reached by using a double crystal Si220 monochromator. Downstream of the monochromator, the beam was focused down to ≈0.6 × 0.8 µm^2^ (vertical × horizontal) using a fixed‐curvature Kirkpatrick–Baez (KB) mirror system. The flux was ≈5 × 10^10^ photons s^−1^. A photodiode collecting the XRF from a thin Ti membrane inserted in the beam path was used to continuously monitor the incoming beam intensity. XRF and scattered radiation were collected with a dispersive energy silicon drift detector (SDD) with an active area of 80 mm^2^ (SGX, Sensortech, Buckinghamshire, UK). Acquisition time per pixel was 100 ms.

##### Nano‐X‐Ray Fluorescence Mapping

Multi‐elemental XRF‐mapping in the nanoscale was performed at the Beamline ID16B of the ESRF.^[^ ^70^ ^]^ The sections previously analyzed at microbeamline ID21 were adhered to kapton tape and fixed on custom‐made XRF‐windows. A pink beam with an energy of 25.6 keV was focused down to ≈60 ×  60 nm² (vertical ×  horizontal) using KB mirrors with a photon density of ≈1 ×  10^11^ photons s^−1^. One seven‐element SDD array (Mirion Technologies Inc., San Ramon, CA, USA) was used to collect XRF. Acquisition time per spectrum was 150 ms dwell time. In contrast to beamline ID21, ID16B does not operate under vacuum conditions and thus does not allow the detection of low Z‐elements. Count rates of XRF‐intensities related to Ca, Ti, V, Cr, Mn, Fe, Co, Ni, Ta, Sr, Zr, Nb, and Mo were computed.

##### Micro‐X‐Ray Absorption Near Edge Structure Analyses

X‐ray XANES spectra were obtained at beamline ID21 as previously described. ^[^ ^71^ ^]^ In brief, for collecting spectra at the Ti K‐edge, the energy of the incoming beam was scanned from 4.95 to 5.0 keV in increments of 0.5 eV, with acquisition times of 100 ms per energy. Five micro‐XANES spectra were collected per point and subsequently averaged and decomposed a through linear combination fitting of XANES spectra of anatase and rutile reference spectra.

##### Isolation and Cultivation of Primary Human Cells

hMSCs, OBs, and BM mononuclear cells (BMMNCs) were isolated from cancellous bone and BM aspirate from a patient who underwent primary implantation of a hip endoprosthesis. In brief, hMSCs were isolated from BM aspirate by plastic adherence after density centrifugation. MSC isolation and characterization was performed as described previously.^[^ ^72^ ^]^ OBs were isolated from diced cancellous bone and characterized as described previously by others.^[^ ^73^ ^]^ The cell culture medium for MSCs and OBs was Dulbecco's modified Eagle's medium with 10% fetal calf serum, 5 m*m* l‐alanyl‐l‐glutamine and 100 U mL^−1^ penicillin plus 100 µg mL^−1^ streptomycin. The cells were cryoconservated in passage two until thawing and expansion to passage four and further use for 3D culture. BMMNCs were isolated from BM aspirate by density centrifugation (Histopaque, 1.077 g mL^−1^) at 400 × *g* for 30 min. After centrifugation, the BMMNCs from the interphase were cryoconservated in RPMI 1640 supplemented with 12.5% human serum albumin and 10% dimethyl sulfoxide. The cells were thawed in RPMI 1640 supplemented with 10% human AB serum and 25 U mL^−1^ nuclease prior to further use for the bone and BM 3D culture.

##### Bone and Bone Marrow‐on‐a‐chip

Emulating in vivo exposure to Co and Cr in vitro was realized by bone and BM 3D cell culture in a microfluidic chip system (HUMIMIC Chip2, TissUse GmbH). The chip design and the protocol regarding culture conditions was adapted from previously described work.^[^ ^63^ ^]^ Modifications of the culture protocol are detailed as follows: Human decellularized cancellous bone^[^ ^74^ ^]^ was used as scaffold material. The cell culture media was supplemented with 10% human AB serum over the whole culture period. 1.5 × 10^5^ human primary mesenchymal stromal cells and 1.5 × 10^5^ OBs were seeded on cylindrical scaffolds (5 mm height, 3 mm radius) before culturing under static conditions in osteoinductive media for 5 days. Osteogenic differentiation was performed as previously described for 2D culture of mesenchymal stromal cells.^[^ ^75^ ^]^ The scaffolds were transferred into the HUMIMIC Chip2 and dynamic microfluidic culture was started (day 0). BMMNCs (1.0 × 10^6^) were seeded on the scaffolds before osteoclastogeneses was induced for 7 days by growth factor supplementation as described in in vitro culture protocols for 2D osteoclast differentiation.^[^ ^76^ ^]^ Growth factor dosimetry was adapted for the 3D culture protocol. Another 2.0 × 10^6^ BMMNCsc were seeded on the scaffold (day 7) before culturing for 20 days without any further growth factor supplementation. Exposure to divalent Co and trivalent Cr was performed as previously described.^[^ ^25^ ^]^ Metal exposure was initiated at day 8 by a media concentration of 2.5 mg L^−1^ Co(II) plus 2.5 mg L^−1^ Cr(III). Exposure was sustained at every media change. After 20 days of dynamic 3D culture (day 28), the scaffolds were fixed and prepared for XRF‐analyses as described for the ex vivo samples.

##### Confocal Fluorescence and Second Harmonic Imaging Microscopy

Confocal and second harmonic microscopy was performed on a Leica SP5 II microscope equipped with a Spectra Physics Multiphoton Laser (Mai Tai HP) using a 25× water immersion objective as described before.^[^ ^77^ ^]^

##### Cytotoxicity Assay

Semiquantitative detection of LDH was performed by using a commercially available cytotoxicity detection kit (Roche) according to the manufacturer's instructions at every media change.

##### Data Presentation and Statistics

XRF‐ and XANES‐spectra were fitted using the software PyMca.^[^ ^78^ ^]^ To assess absolute and relative mass fractions, the spectra of regions allocated to cells were averaged and the peak areas corresponding to individual XRF‐lines were computed into mass fractions, taking into account the matrix composition. The overall density was assumed to be 1.2 g cm^−3^ for intertrabecular soft tissue and 1.9 g cm^−3^ for bone trabeculae.^[^ ^79^ ^]^ Low matrix components were assessed to be 0.5 g (carbon), 0.21 g (oxygen), 0.14 g (hydrogen), and 0.17 g (nitrogen) per cm^3^.^[^ ^80^, ^81^ ^]^ PyMca was used for descriptive statistics regarding RGB imaging, heat maps, and peak spectra. ImageJ was used for the thresholding of the Ca‐maps and for reconstruction of the 3D two‐photon excitation images. GraphPad Prism 8.0.0 was used for statistical analysis and data plotting. No samples or data were excluded from the analysis and all data points are shown as individual values. The Shapiro–Wilk test was used to test normality and the Levene test was used to assess the homogeneity of variances of the data for the indicated groups. Independent and non‐normal distributed data were analyzed using the Mann–Whitney *U*‐test (two sample groups). If normality could be assumed, unpaired Student's t‐test (two sample groups) was chosen to determine statistical significance. Sample size was not predetermined by statistical methods. Analyses of Pearson correlation coefficients were used to assess the linear correlation between metal integration from BM into trabecular bone. All tests were analyzed two‐sided and *p* < 0.05 was regarded as significant. Detailed information about error bars, sample sizes per group, and statistical analysis are included in all figure legends. Randomization was not applied and the investigators were not blinded to group allocation during the experiments because samples from revision surgery often display obvious morphological differences.

## Conflict of Interest

U.M. is shareholder and CEO of TissUse GmbH. B.H. is shareholder and CEO of Xploraytion GmbH. C.P. serves as consultant for Aesculap, CeramTec, Zimmer Biomet, DePuy Synthes, Smith & Nephew, receives royalties from Smith & Nephew, DePuy Synthes, and receives institutional funding and research support from Aesculap. G.N.D. serves as consultant for CeramTec and DePuy Synthes and receives institutional funding and research support from Aesculap, CeramTec, DePuy Synthes, LINK, OHST, Peter Brehm, Smith & Nephew, Zimmer Biomet. These companies did not financially support this study, had no role in study design, sample collection, data collection and analysis, decision to publish, or preparation of the manuscript.

## Supporting information

Supporting Information
