# Supplementary material for: Metal‐Specific Biomaterial Accumulation in Human Peri‐Implant Bone and Bone Marrow
Source: Adv Sci (Weinh). 2020 Aug 3;7(20):2000412. doi: 10.1002/advs.202000412 (PMC7578891; doi:10.1002/advs.202000412)
Supplement: Supplementary file 1 — Supporting Information [file ADVS-7-2000412-s001.pdf]

© 2020 Wiley-VCH GmbH

ADVANCED SCIENCE
Open Access

Supporting Information

for *Adv. Sci.,* DOI: 10.1002/advs.202000412

Metal-Specific Biomaterial Accumulation in Human Peri-Implant Bone and Bone Marrow

Janosch Schoon, Bernhard Hesse, Anastasia Rakow, Melanie J. Ort, Adrien Lagrange, Dorit Jacobi, Annika Winter, Katrin Huesker, Simon Reinke, Marine Cotte, Remi Tucoulou,Uwe Marx, Carsten Perka, Georg N. Duda,* and Sven Geissler*

Supporting Information

Metal-specific biomaterial accumulation in human peri-implant bone and bone marrow

Janosch Schoon, Bernhard Hesse, Anastasia Rakow, Melanie J. Ort, Adrien Lagrange, Dorit Jacobi, Annika Winter, Katrin Huesker, Simon Reinke, Marine Cotte, Remi Tucoulou, Uwe Marx, Carsten Perka, Georg N. Duda**, Sven Geißler*

*carsten.perka@charite.de
**georg.duda@charite.de

Table S1. Baseline patient and implant data

patient	age	sex	revised implant	indication for surgery	surgical treatment	ipsilateral implant status	years since latest ipsilateral surgery	implant status contralateral joint
control 1	70	w	native	knee osteoarthritis	primary TKA	-	-	TKA
control 2	48	w	native	osteoarthritis secondary to hip dysplasia	primary THA	-	-	native
control 3	79	w	native	knee osteoarthritis	primary TKA	-	-	native
control 4	71	w	native	hip osteoarthritis	primary THA	-	-	native
control 5	69	m	native	hip osteoarthritis	primary THA	-	-	THA
control 6	78	m	native	hip osteoarthritis	primary THA	-	-	native
case 1	61	w	modular short stem THA	recurrent dislocation, radiolucent lines at the cup and the modular stem	revision to a elliptic TMT cup with cemented PE inlay, non-modular stem and ceramic head	primary modular short stem THA; cup revision to non-cemented TMT press-fit cup (3 years post-PI)	11	native
case 2	73	m	modular short stem THA	pseudotumor, metallosis	revision to modular TMT cup, PE inlay, ceramic head and non-modular stem; allogeneic bone grafting	primary implantation of a THA; revision to modular short stem prosthesis after fracture of ceramic head (4 years post-PI)	12	THA
case 3	63	w	McMinn HRA implant	pseudotumor, elevated systemic metal levels, metallosis, central acetabular defect	revision to TMT cup, PE inlay, ceramic head and non-modular stem; acetabular augmentation	primary HR implant	12	native
case 4	78	w	McMinn HRA implant	periprosthetic femoral fracture	revision to a modular TMT cup, PE inlay, ceramic head and non-modular stem	primary HR implant	11	THA
case 5	78	m	McMinn HRA implant	progressive pain, acetabular and femoral osteolysis, elevated systemic metal levels	revision to modular TMT cup, PE inlay, ceramic head and non-modular stem; allogeneic bone grafting.	primary HR implant	14	THA

case 6	60	m	McMinn HRA implant	progressive pain, acetabular and femoral osteolysis	revision to modular TMT cup, PE inlay, ceramic head and non-modular stem; allogeneic bone grafting	primary HR implant	12	HR implant
case 7	76	m	MoM THA	pseudotumor, elevated systemic metal levels, osteolysis	revision to elliptic TMT cup with cemented PE inlay, non-modular stem and ceramic head; allogeneic bone grafting	primary HR implant; revision to MoM THA due to femoral neck fracture (first year post-PI)	8	HR implant
case 8	69	w	MoM THA	progressive pain, stem loosening, acetabular osteolysis	revision to elliptic TMT cup, PE inlay, non-modular stem and ceramic head	primary MoM THA	11	native
case 9	61	w	MoM THA	pain, osteolyses, elevated metal levels	revision of the articulating components to ceramic head and PE inlay, allogeneic bone grafting	primary MoM THA	19	THA
case 10	46	m	MoP THA	femoral osteolysis, suspicion of PJI	revision to modular TMT cup, PE inlay, ceramic head and non-modular stem	primary THA; cup revision due to cup fracture (5 years post-PI)	12	native
case 11	58	w	multi-component THA	TMT cup loosening and dislocation, luxation	revision to elliptic TMT cup, PE inlay, CoCrMo head; stem and TMT wedge preserved	primary THA due to osteoarthritis secondary to dysplasia; multiple revisions (6, 14, 22, 25 years post-PI)	9	native
case 12	62	w	multi-component THA	cup loosening, acetabular osteolysis	revision to TMT cup with cup cage construct, PE inlay, ceramic head; stem preserved	primary THA, cup revision(s) (n.a. years post-PI)	n.a.	THA
case 13	75	m	TKA	progressive pain, PE inlay abraded, synovialitis, pseudotumor	cemented hinge TKA	primary TKA	16	native
case 14	71	m	TKA	PE inlay abraded, TKA loosening, metallosis, femoral and tibial osteolysis	cemented hinge TKA	primary TKA	19	native

Abbreviations: CoCrMo, cobalt-chromium-molybdenum alloy; HR, hip resurfacing; MoM, metal-on-metal; MoP, metal-on-polyethylene; n.a.; information not available from clinical records; PE, polyethylene; THA, total hip arthroplasty; TKA, total knee arthroplasty; TMT, trabecular metal technology

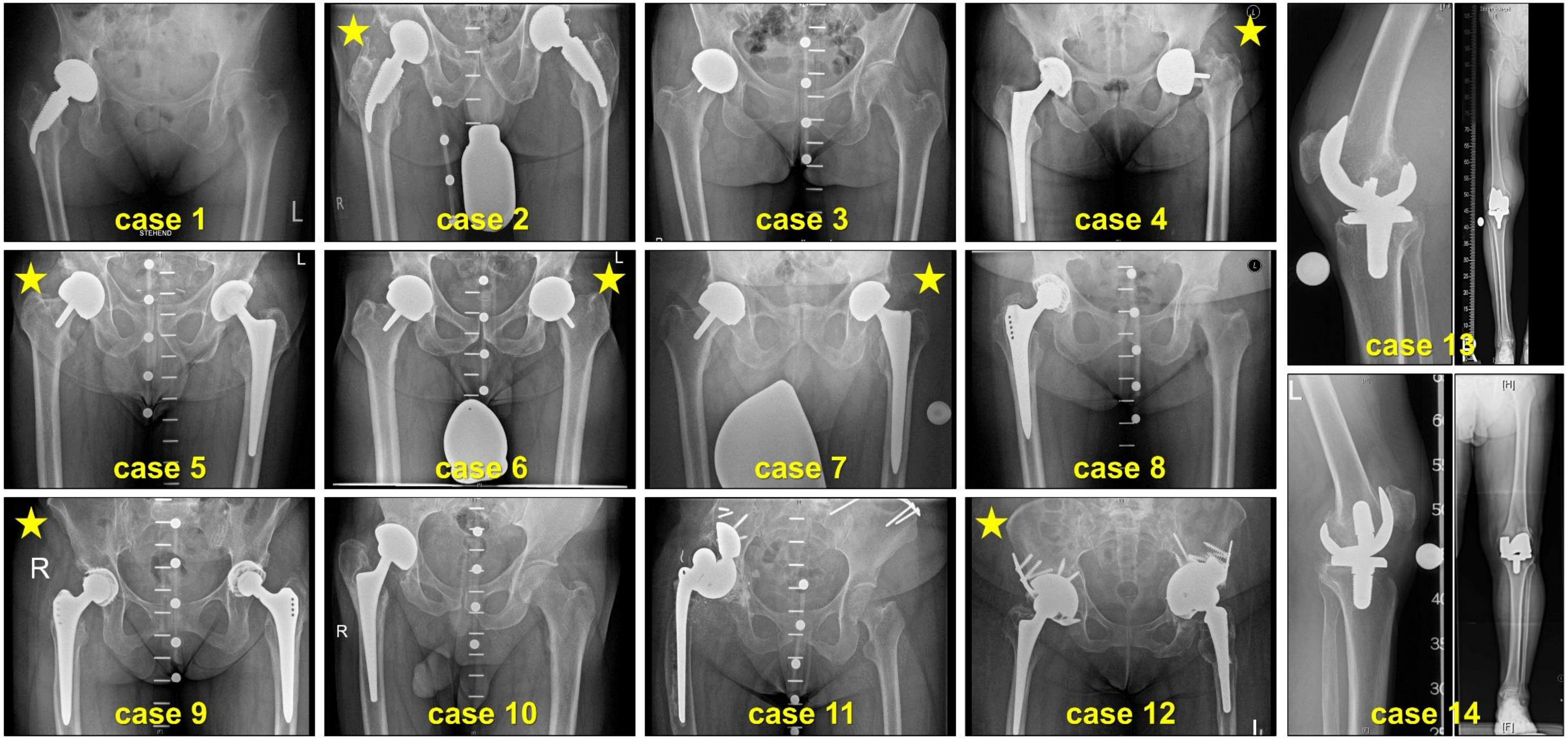

Figure S1. Pre-revisional radiographs of all analyzed cases. In cases of bilateral implants at the time of revision, the yellow asterisk labels the side of the focus implant, i.e. the side of intraoperative surgical sampling.

Table S2. Systemic and local metal levels.

sample ID	serum [µg l^{-1}]		synovial fluid [µg l^{-1}]										
	Co	Cr	Co	Cr	Ti	Al	V	Mn	Ni	Zr	Nb	Mo	Ta
case 1	n.a.	0.7	7.1	6.4	3.2	< 23	< 0.2	2.12	< 6	0.10	< 0.1	0.73	< 0.1
case 2	47.0	15.2	821	659	3.5	11.3	0.42	16.7	18.7	< 0.2	< 0.1	43.1	< 0.1
case 3	171	60.3	6,911	12,298	3.2	< 23	2.09	42.3	13.0	0.49	2.19	367	0.19
case 4	n.a.	n.a.	n.a.	n.a.	n.a.	n.a.	n.a.	n.a.	n.a.	n.a.	n.a.	n.a.	n.a.
case 5	37.3	34.8	3,028	6,967	3.9	< 23	0.52	32.1	9.22	0.18	0.27	301	< 0.1
case 6	2.33[a)]	2.06[a)]	137	194	2.8	< 23	< 0.2	9.62	8.93	0.39	< 0.1	8.93	< 0.1
case 7	16.5	3.0	401	79.4	5.5	< 23	0.21	8.49	< 6	<0 .1	< 0.1	8.68	< 0.1
case 8	n.a.	1.2	19.3	33.8	7.4	< 23	< 0.2	13.3	36.4	0.71	< 0.1	2.85	< 0.1
case 9	n.a.	4.6[b)]	3,487	1,917	12.6	< 23	1.28	26.6	8.11	< 0.1	1.96	154	< 0.1
case 10	n.a.	n.a.	n.a.	n.a.	n.a.	n.a.	n.a.	n.a.	n.a.	n.a.	n.a.	n.a.	n.a.
case 11	< 1	0.60	34.1	22.3	1,316	77.5	64.0	1.20	0.97	3,448	1.14	4.00	6,415
case 12	n.a.	n.a.	n.a.	n.a.	n.a.	n.a.	n.a.	n.a.	n.a.	n.a.	n.a.	n.a.	n.a.
case 13	n.a.	n.a.	12.8	28.6	5.0	< 23	< 0.2	2.32	< 6	0.20	< 0.1	1.85	< 0.1
case 14	n.a.	n.a.	n.a.	n.a.	n.a.	n.a.	n.a.	n.a.	n.a.	n.a.	n.a.	n.a.	n.a.
ref serum	< 0.9	< 0.5	-	-	-	-	-	-	-	-	-	-	-
ref SF[c)]	-	-	< 0.06	< 0.6	< 5	< 23	< 0.2	<1.3	< 2	< 0.2	< 0.1	< 1.75	< 0.1

Abbreviation: n.a., not available; SF, synovial fluid; ref, reference value
[a)]metal quantification in whole blood; [b)]metal quantification one year prior to local sample harvesting in the course of a contralateral revision surgery; [c)]metal quantification in three independent samples of native synovial fluid

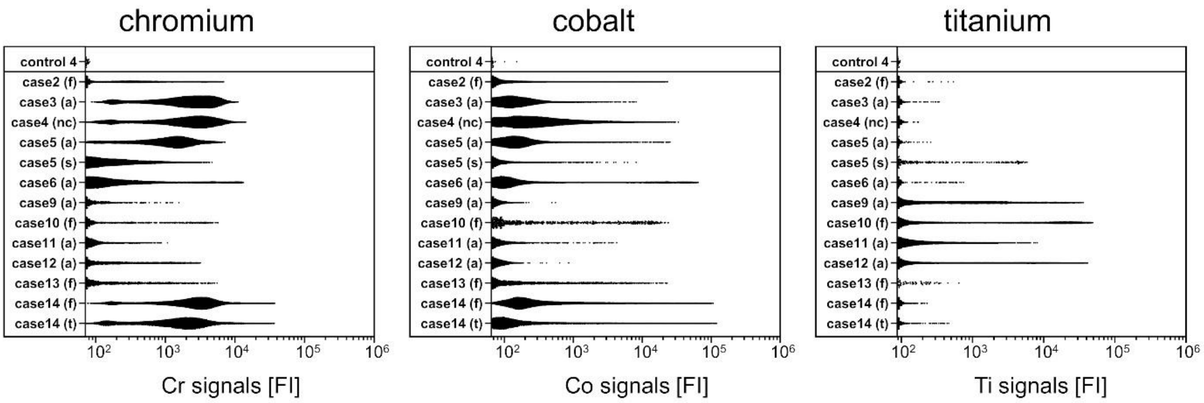

Figure S2. Distributions of the quantitative data points for cobalt, chromium and titanium of all individual nano-XRF maps. Depicted are all data points from maps with 60 nm spatial resolution which are greater than the metal specific 99.9% quantile of the overall data points obtained from the control sample ($Q_{0.999}$ cobalt [FI], 64.50; $Q_{0.999}$ chromium [FI], 70.74; $Q_{0.999}$ titanium [FI], 87.16). Abbreviations: FI, fluorescence intensity

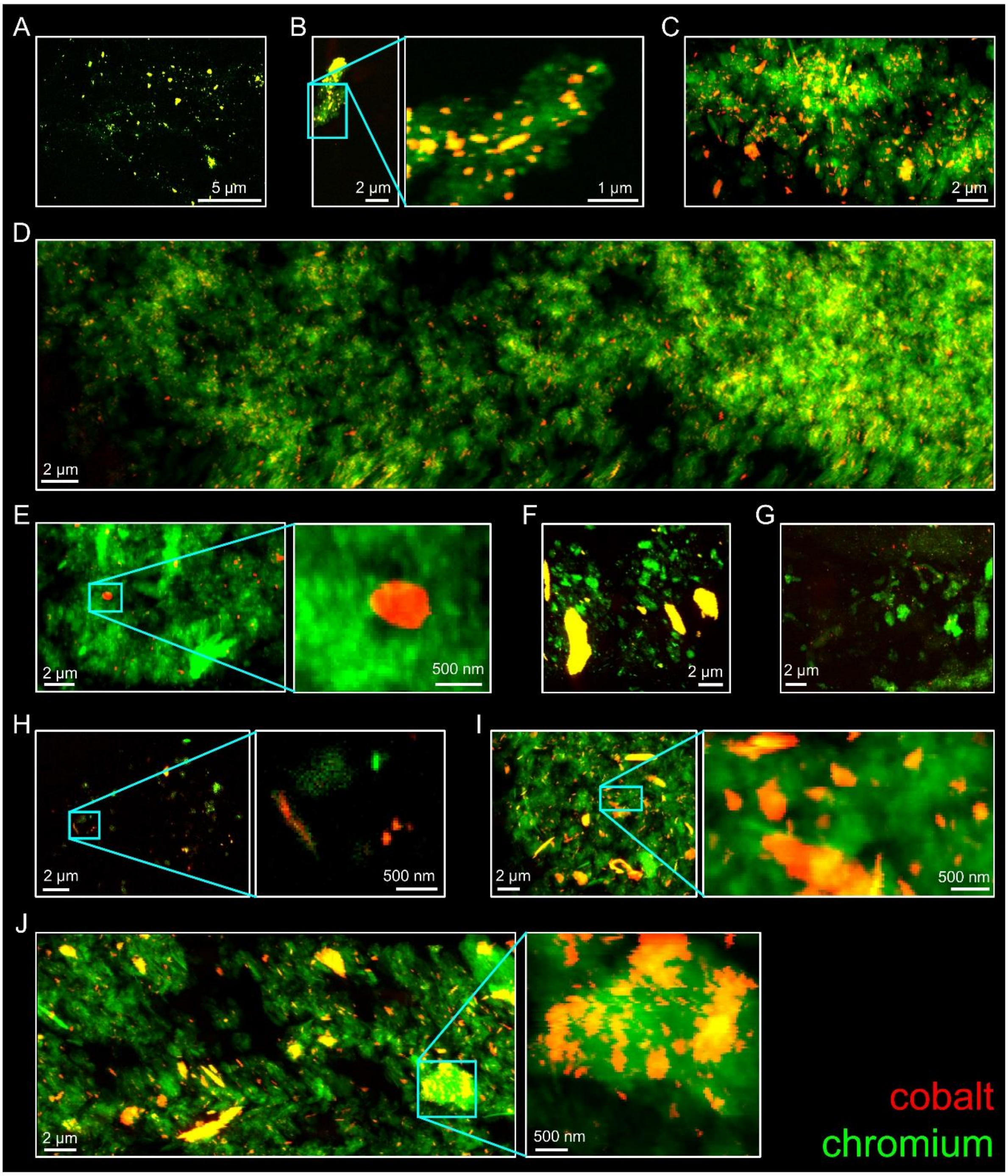

Figure S3. XRF-scans at 60 nm and 30 nm resolution of peri-implant bone marrow specimens show substantial exposure to CoCr containing micron-, submicron- and nanoparticles. Cobalt is exclusively co-localized with chromium and present in the particulate state. Chromium is also present without co-localized cobalt in characteristic dissolution clouds. Depicted are qualitative images of the XRF-scans from various implant types: (A, B) modular stem total hip arthroplasty implant, (C-G) hip resurfacing implants, (H) revision total hip arthroplasty implant and (I, J) total knee arthroplasty implant.

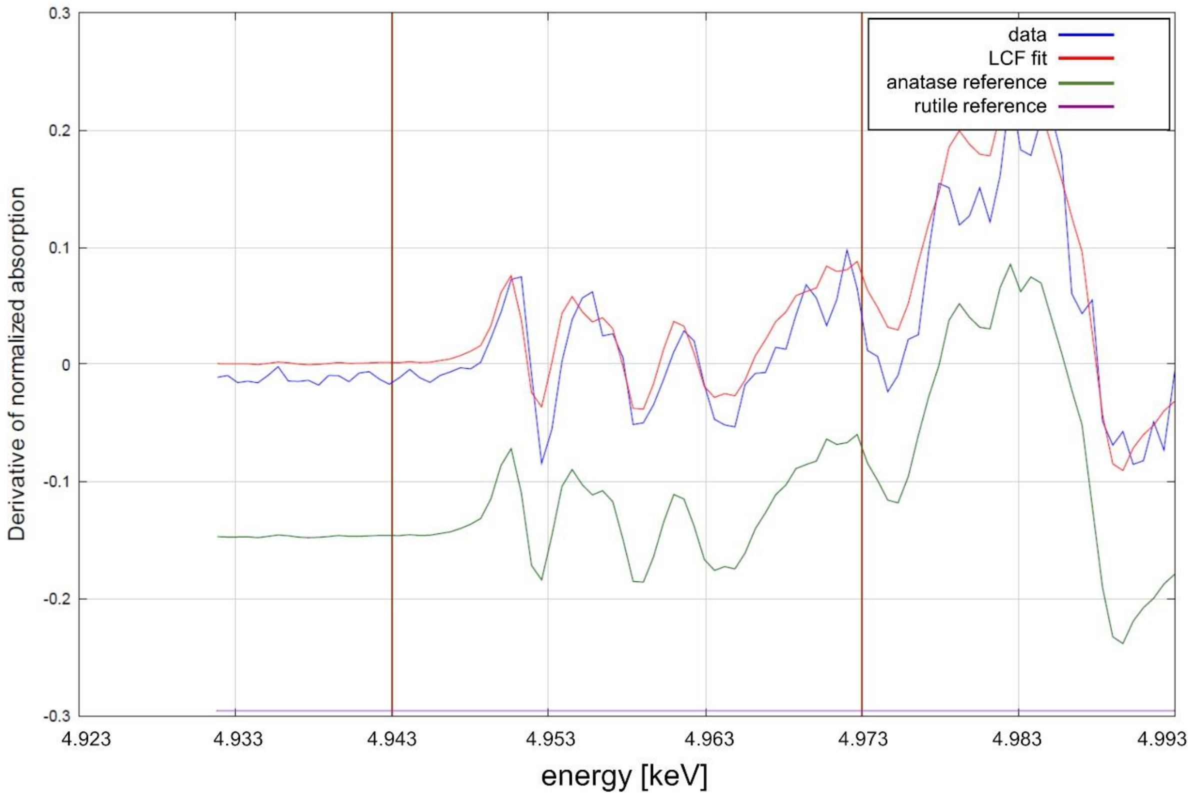

Figure S4. Linear combination fitting of X-ray absorption near edge structure analyses indicate predominate titanium dioxide in the anatase crystal phase of the analyzed Ti-containing particle. Plotted are the first derivatives of the absorption values. Sum spectrum of n = 5 analyzed particles (data) in comparison to reference spectra of rutile and anatase. The linear combination fitting reveals that no contribution of rutile is required for the best linear combination fit.

Table S3. Detailed information on chemicals, buffers and equipment used in the course of sample harvesting and preparation.

Chemicals, buffers & equipment	Trade name	Manufacturer
4% Paraformaldehyde solution	(prepared from) 16% Paraformaldehyde aqueous solution	Electron Microscopy Sciences
Phosphate-buffered saline	Dulbecco's Phosphate-Buffered Saline	Gibco
Embedding cassettes	Tissue-Tek Mega-Cassettes	Sakura
Ethanol of various concentrations	(prepared from) Ethanol ≥ 99.5 %	Carl Roth
Xylene	Xylenes, Histological Grade	Fisher scientific
Methyl methacrylate	Technovit 9100 liquid	Kulzer
Benzoyl peroxide / dicyclohexyl phthalate	Technovit 9100 hardener 1	Kulzer
Poly(methyl methacrylate)	Technovit 9100 powder	Kulzer
3,5,N,N-tetramethylaniline	Technovit 9100 hardener 2	Kulzer
1-Decanethiol	Technovit 9100 regulator	Kulzer
Rotary microtome	Rotary microtome RM 2255	Leica
Tungsten carbide blade	TC knife profile d,16 cm,40,50,60 degrees	Leica
Ultralene microfoil	Pre-Cut Circular Window Film	SPEX SamplePrep
Polyethylene foil	Kisol foil	Kettenbach
2-Methoxyethyl acetate	2-Methoxyethyl acetate for synthesis	Merck Millipore
DMEM	Dulbecco's modified Eagle's medium - low glucose	Sigma-Aldrich
Fetal calf serum	FBS Superior	Biochrom
L-alanyl-L-glutamine	GlutaMAX	Gibco
Penicillin / Streptomycin	Penicillin 10.000 U ml^{-1} / Streptomycin 10.000 µg ml^{-1}	Biochrom
Histopaque, 1.077 g ml^{-1}	Histopaque®-1077	Sigma-Aldrich
Human serum albumin	ALBUNORM 20%	Octapharma
RPMI 1640	RPMI-1640 Medium with L-glutamine and sodium bicarbonate	Sigma-Aldrich
Human AB serum	Serum from human male AB plasma	Sigma-Aldrich
Nuclease	Pierce Universal Nuclease for Cell Lysis	Thermo Fisher
